# Interpreting Liver Function Abnormalities in Returned Travelers: Clinical Context Matters More Than Pathogen-Specific Signatures

**DOI:** 10.7759/cureus.109184

**Published:** 2026-05-19

**Authors:** Bouchra Lachhab, Perry J van Genderen

**Affiliations:** 1 Department of Internal Medicine, Harbour Hospital and Institute for Tropical Diseases, Rotterdam, NLD; 2 Expertise Centre Travel Risk Management, Corporate Travel Clinic, Rotterdam, NLD

**Keywords:** abnormalty, enzymes, etiologic, injury, liver, syndromic, traveler

## Abstract

Liver function test abnormalities (LFTA) are frequently observed in ill-returned travelers, yet their diagnostic value remains uncertain. To gain more insights, a retrospective cohort study of 2,957 consecutive ill-returned travelers presenting to a national reference center for tropical diseases between 2012 and 2015 was conducted. The associations between clinical presentation, broad etiologic categories (viral, bacterial and parasitic infections) and the presence, severity and type of LFTA (parenchymatous, cholestatic or mixed) were evaluated using chi-square analysis, Cramér’s V and standardized residuals. Pathogen-level analyses assessed variability in the frequency and severity of liver involvement.

LFTA were present in 40% of patients and were predominantly mild with a mixed biochemical pattern. Clinical presentation was strongly associated with the presence, severity and type of LFTA. Febrile illness was associated with a higher likelihood of LFTA and with more severe and mixed-type abnormalities, whereas gastrointestinal and cutaneous presentations were associated with milder abnormalities. Broad etiologic categories were associated with the presence and severity of LFTA but showed no clinically meaningful association with biochemical pattern. Pathogen-level analyses revealed substantial heterogeneity in liver involvement.

LFTA were prevalent but mainly non-specific in returning travelers. Clinical presentation offered more diagnostic insight than classification in viral, bacterial and parasitic etiology when interpreting liver involvement. LFTA should be evaluated within the clinical context, rather than as specific indicators of pathogens.

## Introduction

International travel is frequently associated with illness, affecting a substantial proportion of travelers [1-3]. Among individuals presenting with travel-related illness, abnormalities in liver biochemistry are commonly observed, irrespective of systemic inflammatory markers [4].

These abnormalities may arise from infections that directly affect hepatic tissue, such as hepatotropic viruses, or from systemic infections in which liver injury occurs secondary to inflammatory or toxic mechanisms [5]. In addition, a broad range of non-infectious conditions - including metabolic disease, alcohol-related liver injury, cardiovascular disorders, and drug-induced hepatotoxicity - may contribute to abnormal liver enzymes [5,6].

A large, controlled, cross-sectional study of more than 14,000 returned travelers demonstrated that elevations in transaminases such as aspartate aminotransferase (AST) and alanine aminotransferase (ALT) are significantly associated with imported infectious diseases, supporting their clinical relevance as markers of infection. In that study, elevated transaminase values were linked to a broad range of viral, bacterial and protozoan infections, whereas gamma-glutamyl transferase (GGT) did not provide additional diagnostic value [4]. However, this study primarily focused on the presence of enzyme abnormalities across specific infectious diagnoses and did not evaluate the relative diagnostic value of clinical presentation versus etiologic classification in predicting liver involvement.

The primary objective of this study was to determine the association between clinical presentation and broad etiologic categories with the presence of liver function test abnormalities (LFTA) in ill-returned travelers. The secondary objectives were to assess associations with the severity and biochemical pattern of LFTA and to explore pathogen-level variability in liver involvement.

## Materials and methods

Study design and population

A retrospective single-center cohort study was conducted, which included 2,957 consecutive ill-returned travelers presenting to a national reference center for tropical diseases between 2012 and 2015. Duplicate visits were excluded where identifiable. Patients with pre-existing (chronic) liver disease, malignancy, pregnancy, or age <16 years were excluded.

Clinical and etiologic classification

Clinical syndrome categorization was based on the clinical assessment documented at presentation and verified during retrospective chart review. Clinical presentations were grouped into five syndromic categories: fever, gastrointestinal tract, respiratory tract, skin and others. Patients with overlapping symptoms were classified according to the dominant clinical syndrome documented at presentation. Infectious etiologies were classified as viral, bacterial or parasitic infections. Patients with uncertain diagnoses, co-infections or non-infectious diagnoses were excluded from etiologic subgroup analyses but retained in overall analyses where appropriate. Diagnostic confirmation was based on standard clinical microbiological methods available during the study period, including serology, polymerase chain reaction (PCR), microscopy, culture and antigen testing depending on the suspected pathogen and clinical presentation.

Laboratory investigations

Laboratory analyses were performed using standard hematological and biochemical tests on peripheral blood samples. Reference values are shown in Table 1.

**Table 1:** Gender-specific reference values of the laboratory parameters

Parameter	Normal value (females)	Normal value (males)
Aspartate Aminotransferase, AST (U/L)	<30	<35
Alanine Aminotransferase (ALT) (U/L)	<35	<45
Gamma-Glutamyl Transferase (GGT) (U/L)	<40	<55
Alkaline Phosphatase (ALP) (U/L)	<100	<115
Total Bilirubin (μmol/L)	<17	<17
C-reactive Protein (CRP) (mg/L)	<10	<10
Leukocytes (×10⁹/L)	<10	<10

Characterization of liver function test abnormalities

Severity of LFTA

Liver function test results were expressed as a ratio relative to the upper limit of normal (ULN). Values >1× ULN were considered abnormal and classified according to a modified WHO toxicity grade system (grades 0 to III) as shown in Table 2.

**Table 2:** Gradation of liver function test abnormalities (LFTA) on admission Liver function test abnormalities were classified according to a modified WHO toxicity grade system [7]. ULN: Upper limit of normal.

Class	Grade	Definition
No LFTA	0	≤1.0 X ULN
Mild LFTA	I	≤2.5 × ULN
Moderate LFTA	II	>2.5 to ≤5.0 × ULN
Severe LFTA	III	>5.0 × ULN

Biochemical Enzyme Pattern of Liver Function Test Abnormalities

Biochemical enzyme patterns of liver function test abnormalities were classified as three distinctive patterns as shown in Table 3.

**Table 3:** Classification of enzyme patterns of liver function test abnormalities (LFTA) Note: Bilirubin and lactate dehydrogenase (LDH) were excluded from enzyme pattern classification due to potential confounding by hemolysis. AST: Aspartate aminotransferase; ALT: alanine aminotransferase; GGT: gamma-glutamyl transferase

Biochemical pattern	Definition
Parenchymal pattern	Predominant ALT/AST elevation
Cholestatic pattern	Predominant ALP/GGT elevation
Mixed pattern	No clear predominance

Hepatic Involvement Profile of a Pathogen

Hepatotropism of a pathogen was defined as the proportion of patients exhibiting LFTA among all individuals infected with a given pathogen.The grading of LFTA was used as a proxy measure for the severity of liver involvement.

Statistical analysis

Missing data were handled by complete-case analysis for each comparison. Because of the retrospective aggregated design and incomplete availability of covariates, multivariable modeling was not performed. Although multivariable analysis could theoretically strengthen adjustment for confounding, the aggregated retrospective dataset and substantial missingness in selected covariates limited the reliability of adjusted modeling.

Data were presented as percentages, mean with standard deviation, or median with interquartile range (IQR) using descriptive statistics. Differences between groups were analyzed by either t-test, Mann-Whitney U test or chi-square as appropriate. Associations between categorical variables were assessed using chi-square tests of independence. Effect sizes were quantified using Cramér’s V to assess the strength of associations. For each contingency table, standardized residuals were calculated to identify over- and underrepresented combinations and visualized using mosaic plots. Odds ratios (ORs) with 95% confidence intervals (CIs) were computed for selected comparisons to quantify the strength of associations. To further explore the structure of relationships between variables, correspondence analysis was performed, allowing visualization of row and column profiles in a reduced-dimensional space. The proportion of variance (inertia) explained by each dimension was used to interpret the relative contribution of each axis to the overall association. All analyses were conducted using aggregated data, and results are therefore presented as unadjusted estimates. Pathogen-level analyses were performed using descriptive measures of hepatotropism and severity of liver involvement.

## Results

Overall findings

LFTA were present on admission in 40% of 2,957 ill-returning patients. As shown in Table 4, patients with LFTA were significantly older and more frequently men. In addition, significant differences were observed in the proportion of patients with overweight and medication use (including those with a potential hepatotoxic effect). Further, significant differences were observed between patients with LFTA and without LFTA on admission regarding the continent visited, in particular patients with LFTA traveled more frequently to Africa and less frequently to Asia than patients without LFTA.

**Table 4:** General characteristics of 2,957 ill-returned travelers, grouped by presence or absence of liver function test abnormalities (LFTA) on admission AST: Aspartate aminotransferase; ALT: alanine aminotransferase; GGT: gamma-glutamyl transferase; ALP: alkaline phosphatase; IQR: interquartile range. ^(1)^ Mann-Whitney U test; U=897762; Z=-6,6; missing values n=0 ^(2)^ χ2 test; χ=5,84; missing values n=1 ^(3)^ χ2 test; χ=30,16; missing values n=1,866 ^(4)^ χ2 test; χ=9,18; missing values n=0 ^(5)^ χ2 test; χ=10,38; missing values n=0 ^(6)^ Mann-Whitney U test; U=7153162; Z=-11,6; missing values n=126 ^(7)^ Mann-Whitney U test; U=985440; Z=-1,1; missing values n=52 ^(8)^ Mann-Whitney U test; U=285360; Z=-32,3; missing values n=86 ^(9)^ Mann-Whitney U test; U=329223; Z=-31,1; missing values n=29 ^(10)^ Mann-Whitney U test; U=331351; Z=-29,4; missing values n=133 ^(11)^ Mann-Whitney U test; U=574705; Z=-18,9; missing values n=81 ^(12)^ Mann-Whitney U test; U=838812; Z=-2,6; missing values n=226 ^(13)^ χ2 test for trend; χ=6,61; missing values n=55

	LFTA (n=1,174)	No LFTA (n=1,783)	p-value
Demographic parameters			
Age (yrs), median (IQR)	43 (28-54)	35 (26-50)	<0.0001 ^(1)^
Gender, n (%)			0.0157 ^(2)^
Male	594 (50.6%)	822 (46.1%)	
Female	579 (49.4%)	961 (53.9%)	
Overweight (BMI>25 kg/m^2^), n (%)	168 (43.0%)	187 (26.7%)	<0.0001 ^(3)^
Medication use, n (%)	414 (35.3%)	534 (29.9%)	0.0025 ^(4)^
Potential hepatotoxic medication, n (%)	315 (76.1%)	355 (66.5%)	0.0013 ^(5)^
Laboratory parameters on admission			
CRP (mg/L), median (IQR)	9 (2-40)	3 (1-16)	<0.0001 ^(6)^
Leukocytes (x 10^9^/L), median (IQR)	7 (5-9)	7 (6-8)	0.2858 ^(7)^
AST (U/L), median (IQR)	34 (25-46)	21 (18-24)	<0.0001 ^(8)^
ALT (U/L), median (IQR))	38 (25-62)	19 (14-25)	<0.0001 ^(9)^
GGT (U/L), median (IQR)	47 (25-78)	18 (13-26)	<0.0001 ^(10)^
ALP (U/L), median (IQR)	79 (64-101)	64 (54-76)	<0.0001 ^(11)^
Bilirubin (umol/L), median (IQR)	8 (5-12)	8 (5-11)	<0.0083 ^(12)^
Destination (continent of exposure)			0.0101 ^(13)^
Asia, n (%)	390 (33.7%)	770 (44.1%)	
Africa, n (%)	496 (42.9%)	608 (34.8%)	
Australia/Oceania, n (%)	10 (0.9%)	7 (0.4%)	
Europe, n (%)	51 (4.4%)	40 (2.3%)	
North America, n (%)	72 (6.2%)	144 (8.3%)	
South America, n (%)	138 (11.9%)	176 (10.1%)	

Clinical presentation by post-travel syndrome

Presence of LFTA

**Figure 1:**
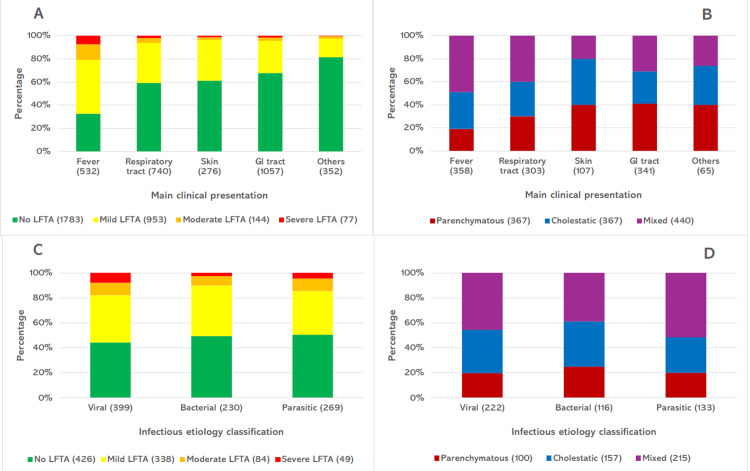
Liver function test abnormalities (LFTA) by clinical presentation (syndrome) and infectious etiology category. (A) Frequency and severity of LFTA in post-travel syndromes, categorized as none (green), mild (yellow), moderate (orange), and severe (red). (B) Distribution of LFTA patterns in post-travel syndromes: parenchymatous (red), cholestatic (blue), and mixed (purple). (C) Frequency and severity of LFTA by infectious etiology (viral, bacterial, parasitic), using the same severity scale as in (A). (D) Distribution of LFTA patterns by infectious etiology, using the same categories as in (B). Number of patients in each category are shown in parentheses.

As shown in Figure 1, majority of the LFTA were mild (81.1%) and biochemical pattern of LFTA varied, with a slight predominance (37.5%) of mixed biochemical enzyme patterns. As shown in Figure 1A, clinical presentation was strongly associated with the presence of LFTA (χ²(4)=260.5, p<0.001; Cramér’s V=0.30). Febrile illness was markedly associated with LFTA (odds ratio (OR) 4.06, 95% confidence interval (CI) 3.32-4.95), whereas gastrointestinal (OR 0.61, 95% CI 0.52-0.71) and other syndromes (OR 0.31, 95% CI 0.23-0.40) were less frequently associated. No associations were observed for respiratory or dermatological syndromes.

Severity of LFTA

**Figure 2:**
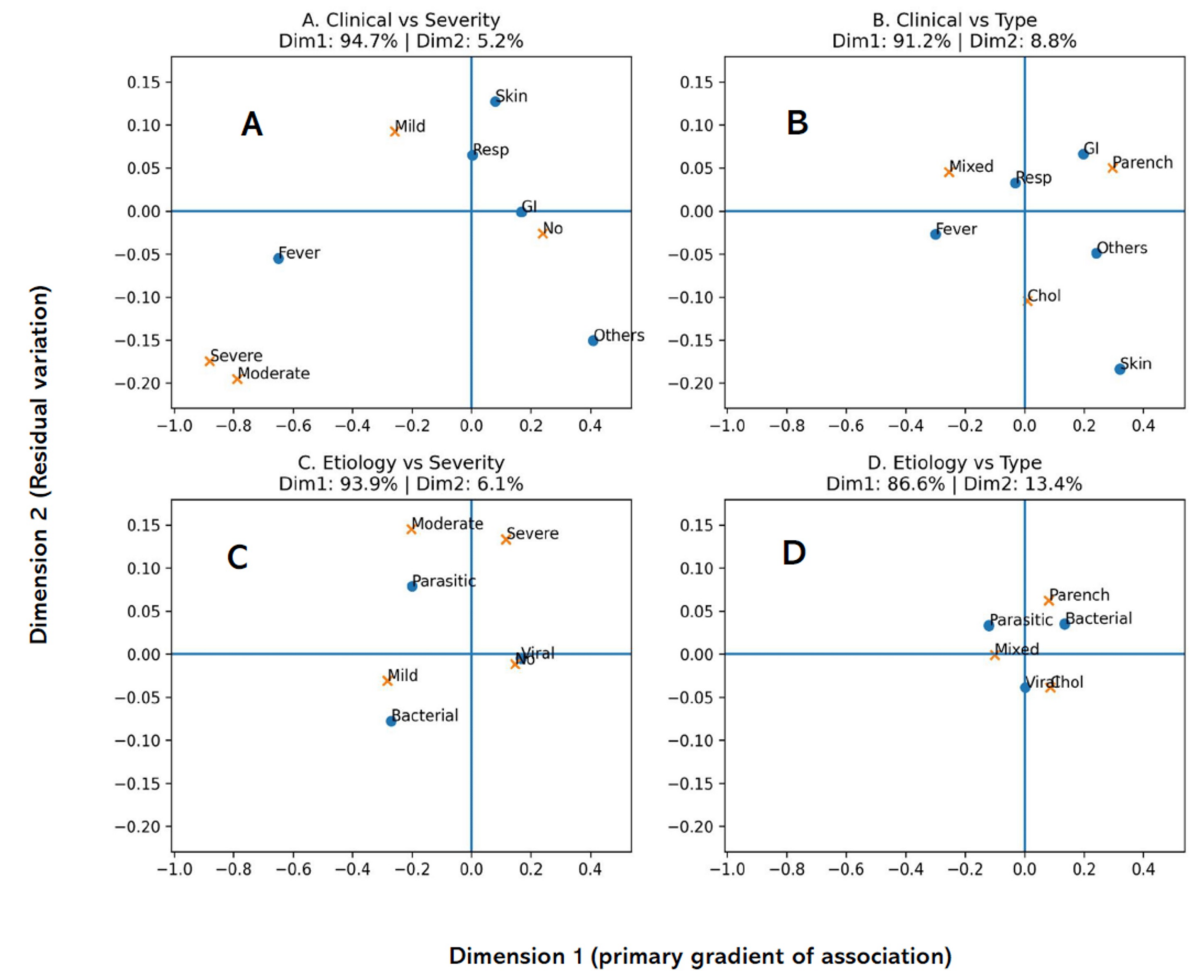
Correspondence analysis of clinical presentation and etiologic class in relation to severity and type of liver function test abnormalities (LFTA). (A) Clinical presentation vs LFTA severity. (B) Clinical presentation vs LFTA type. (C) Etiologic class vs LFTA severity. (D) Etiologic class vs LFTA type. In each panel, row categories are represented by circles and column categories by crosses; proximity between points reflects the strength of association. The horizontal axis (Dimension 1) represents the primary pattern of association, while the vertical axis (Dimension 2) captures secondary variation. Coordinates may be negative or positive, indicating positions on opposite sides of the association pattern and interpreted relative to one another. In panels A and C, Dimension 1 reflects a gradient from absent or mild to increasing severity of LFTA. In panel B, Dimension 1 separates parenchymal from mixed biochemical patterns. In panel D, no clear structure is observed, consistent with the absence of a meaningful association between etiologic class and LFTA type.

As shown in Figure 2A, correspondence analysis showed a clear gradient from absence to increasing severity of LFTA, with the first dimension explaining 94.7% of the variance. Febrile illness clustered with moderate and severe LFTA, whereas gastrointestinal syndromes clustered with absence or mild abnormalities. Respiratory and dermatological syndromes showed no clear association.

Biochemical Pattern of LFTA

Among patients with LFTA, clinical presentation was associated with LFTA type (χ²(8)=66.5, p<0.001; Cramér’s V=0.17) as shown in Figure 1B. Febrile illness was associated with mixed biochemical patterns, whereas gastrointestinal and dermatological syndromes were associated with parenchymal abnormalities. Correspondence analysis (Figure 2B) confirmed a remarkable separation between mixed and parenchymal patterns (dimension 1: 91.2% variance).

Etiologic categories

Presence of LFTA

When limiting the analysis to patients with a known infectious etiology, LFTA were present in 471 of 897 (52.5%) patients. As shown in Figure 1C, etiologic class was associated with the presence of LFTA (χ²(2)=67.3, p<0.001; Cramér’s V≈0.18). Compared with viral infections, LFTA were more frequent in parasitic (OR 1.71, 95% CI 1.30-2.25) and bacterial infections (OR 1.76, 95% CI 1.32-2.35).

Severity of LFTA

LFTA severity differed across etiologic classes (χ²(6)=49.9, p<0.001). Compared with viral infections, parasitic (OR 2.10, 95% CI 1.58-2.80) and bacterial infections (OR 2.19, 95% CI 1.61-2.96) were associated with higher odds of LFTA. Mosaic and correspondence analyses (Figure 2C) showed that viral infections clustered with absence of LFTA, parasitic infections with moderate-to-severe abnormalities and bacterial infections with mild abnormalities (dimension 1: 93.9% variance).

Biochemical Pattern of LFTA

No association was observed between etiologic class and LFTA type (χ²(4)=4.67, p=0.32; Cramér’s V=0.07) (Figure 1D). Mosaic and correspondence analyses showed no meaningful clustering between etiologic categories and biochemical patterns (Figure 2D).

Pathogen-level analysis

**Figure 3:**
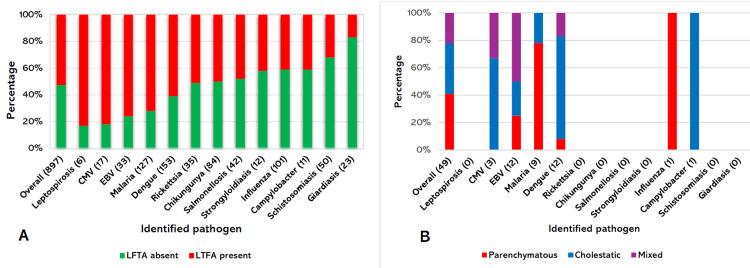
Liver function test abnormalities (LFTA) by identified pathogen. (A) Proportion of patients with and without LFTA across specific pathogens. Bars show LFTA absent (green) and present (red). (B) Distribution of only *severe* LFTA patterns on admission by pathogen, categorized as parenchymatous (red), cholestatic (blue), and mixed (purple). Number of patients in each category are shown in parentheses.

To explore pathogen-specific effects, analyses were performed for selected infectious agents (Figure 3). Hepatotropism varied widely between pathogens, ranging from 17% in Giardia infections to over 80% in infections such as leptospirosis and cytomegalovirus. The severity of liver involvement among patients with LFTA also differed substantially. Epstein-Barr virus infection showed the highest frequency of severe liver involvement (48%), followed by cytomegalovirus (21%), dengue (13%) and malaria (10%), whereas most other pathogens were associated with little or no severe liver involvement. These findings highlight marked heterogeneity at the pathogen level, which is not captured by following broad etiologic categories. The biochemical patterns of severe LFTA differed substantially between pathogens (predominantly parenchymatous in malaria and cholestatic in dengue and cytomegalovirus; all three biochemical patterns occurred in Epstein-Barr virus infections).

## Discussion

In the context of returned travelers, LFTA may result from a wide range of infectious etiologies as well as non-infectious causes such as drug-induced liver injury from travel-related medications. The challenge for the clinician lies in interpreting these biochemical patterns in the context of travel history, geographic exposure, incubation periods and clinical presentation to arrive at an accurate diagnosis [8].

While the hepatotoxic potential of certain pathogens has clearly been documented in case reports and case series describing a broad array of travel-related infections like malaria [9], tick-borne relapsing fever [10], scrubtyphus [11], hepatitis A [12], dengue [13], combined infections [14, 15], leptospirosis [16], typhoid fever [17], amebic abscess [18], visceral leishmaniasis [19], hepatitis C [20], babesiosis [21], brucellosis [22], Q fever [23] as well as non-communicable causes like substance abuse of kava [24], still little is known about the prevalence and severity of LFTA in ill-returning travelers upon admission in a real-world setting and how to interpret them.

The landmark controlled cross-sectional study of 14,559 German travelers returning from (sub)tropical regions found that ALT elevations occurred in approximately 13% of symptomatic returned travelers, while AST elevations were present in approximately 8% [4]. GGT elevations were of limited diagnostic utility. However, this study primarily focused on the presence of isolated enzyme abnormalities across specific infectious diagnoses, whereas clinical evaluation of ill-returned travelers usually starts with the signs and symptoms on admission, conveniently clustered in so-called post-travel syndromic presentations [2]. In addition, it is not always possible to identify a causative pathogen. For example, in the current cohort of 2,957 ill-returned travelers referred to an expertise center, a causative pathogen could be identified in only 30% of the cases.

With a post-travel syndromic presentation-based approach, LFTA appeared common, occurring in 40% of patients, and were predominantly mild with a slight predominance of mixed biochemical patterns. These findings confirm that liver involvement is a frequent but largely non-specific feature of travel-related illness, and extend previous observations by providing a structured framework for interpreting these abnormalities in clinical practice [5].

Interestingly, the clinical presentation showed stronger associations of liver involvement, especially regarding the presence and severity of LFTA. In particular, febrile illnesses were strongly correlated with a higher frequency and greater severity of LFTA, while gastrointestinal and dermatological presentations were linked to milder or absent abnormalities. Interestingly, patients with LFTA had more frequently visited Africa, whereas patients without LFTA had more frequently visited Asia. In line with the observations of others [2,3] but still speculatively, ill-returned patients from sub-Saharan Africa tend to present more commonly with febrile syndromes, whereas gastrointestinal syndromes (acute and chronic diarrhea and non-diarrheal illnesses) are more likely in ill-returned patients from Asia.

The classification in viral, bacterial and parasitic etiologies provided additional, yet more limited, insights. Parasitic and bacterial infections were associated with a higher likelihood and increased severity of LFTA compared to viral infections, suggesting that pathogen type may be associated with the degree of liver injury. However, this classification did not differentiate between the biochemical patterns of liver damage. This distinction is crucial: while etiology influences the severity of liver involvement, it does not dictate how it presents biochemically.

Conversely, the biochemical pattern of liver injury was more closely associated with clinical presentation than with etiological classification. Febrile illnesses were linked to mixed biochemical patterns, while gastrointestinal and dermatological syndromes correlated with parenchymal abnormalities. The lack of a connection between the etiological class and biochemical patterns suggests that these patterns are not specific signatures of pathogens but may instead reflect broader host or systemic processes.

Importantly, clinical presentation consistently demonstrated stronger associations with liver involvement than etiologic grouping, as reflected by larger Cramér’s V effect sizes across analyses. This difference was particularly evident for biochemical patterns of LFTA, where clinical presentation showed a modest but meaningful association (Cramér’s V=0.17), whereas etiologic grouping demonstrated only minimal association (Cramér’s V=0.07). These quantitative differences support the notion that clinical context provides greater interpretive value than broad pathogen classification when evaluating LFTA in returned travelers.

At the pathogen level, significant variability was noted. The potential for hepatotropism and severity of liver involvement differed greatly among infections, with pathogens like Epstein-Barr virus (EBV), cytomegalovirus (CMV), dengue, and malaria demonstrating a higher likelihood or severity of liver involvement, whereas others exhibited minimal hepatic effects, in line with the observations of others [4,8]. These observations suggest that broad etiological categories may obscure clinically significant differences between pathogens within the same category and that liver injury likely results from the complex interplay of pathogen-specific effects like hepatotropism, hepatotoxicity and pathogen load on one hand and the strength and quality of host-defense response on the other hand.

In summary, these findings advocate for a clinically oriented approach where LFTA are primarily interpreted in light of the presenting syndrome. The clinical presentation showed stronger associations of liver involvement, especially regarding the presence and severity of LFTA. Severe LFTA were particularly seen in EBV, CMV, dengue and malaria infections, but were present in association with only 10% of the identified pathogens. Biochemical patterns of LFTA varied widely across pathogens and were of limited predictive value.

Limitations

Several limitations should be considered. First, etiologic analyses were based on broad categories, which may have obscured pathogen-specific effects; however, this reflects clinical reasoning at presentation and enhances applicability. Second, etiologic diagnoses were available for only a subset of patients, introducing potential selection bias. Third, the cross-sectional design precludes assessment of the temporal evolution of LFTA. Fourth, the study was conducted in a national referral center for tropical diseases, which may have enriched the cohort for patients with more severe, complex or diagnostically challenging illnesses. Consequently, the findings may not be fully generalizable to primary care settings or general travel clinics. Five, the retrospective aggregated dataset and substantial missingness in selected covariates like BMI limit the reliability of adjusted multivariable analyses. All findings were therefore interpreted as unadjusted associations. As a result residual confounding - for instance by differences in co-morbidities and/or medication use - cannot be excluded. Finally, although the data were collected between 2012 and 2015 and travel epidemiology has evolved since then, interpretation of liver enzyme abnormalities in returned travelers remains a clinically relevant diagnostic challenge. The current findings are likely to remain relevant in patient care.

## Conclusions

LFTA are common but largely non-specific in returned travelers. Clinical presentation and etiologic class both influence the severity of liver involvement, whereas the biochemical pattern is primarily determined by clinical presentation and not by etiologic grouping. These findings suggest that liver function test abnormalities should be interpreted within the clinical context rather than as pathogen-specific signatures, particularly in the early diagnostic evaluation on admission.
